# Use of lung ultrasound in school-aged children with wheezing

**DOI:** 10.3389/fped.2022.926252

**Published:** 2023-01-09

**Authors:** Marina Attanasi, Simone Sferrazza Papa, Annamaria Porreca, Giuseppe F. Sferrazza Papa, Paola Di Filippo, Francesca Piloni, Giulia Dodi, Francesco Sansone, Sabrina Di Pillo, Francesco Chiarelli

**Affiliations:** ^1^ Department of Pediatrics, Pediatric Allergy and Pulmonology Unit, University of Chieti-Pescara, Chieti, Italy; ^2^ Department of Economic Studies, University of Chieti-Pescara, Chieti, Italy; ^3^ Department of Health Sciences, Università degli Studi di Milano, Milan, Italy; ^4^ Department of Neurorehabilitation Sciences, Casa di Cura del Policlinico, Milan, Italy

**Keywords:** lung ultrasound, children, wheezing, respiratory tract infections, pneumonia

## Abstract

**Background:**

There is limited information available on fast and safe bedside tools that could help clinicians establish whether the pathological process underlying cases of wheezing is due to asthmatic exacerbation, asthmatic bronchitis, or pneumonia. The study's aim was to characterize Lung Ultrasound (LUS) in school-aged children with wheezing and evaluate its use for their follow-up treatment.

**Materials and methods:**

We carried out a cross-sectional study involving 68 consecutive outpatients (mean age 9.9 years) with wheezing and suggestive signs of an acute respiratory infection. An expert sonographer, blinded to all subject characteristics, clinical course, and the study pediatrician's diagnosis, performed an LUS after spirometry and before BDT. The severity of acute respiratory symptoms was determined using the Pediatric Respiratory Assessment Measure (PRAM) score.

**Results:**

The LUS was positive in 38.2% (26/68) of patients [12 (46.1%) with multiple B-lines, 24 (92.3%) with consolidation, and 22 (84.6%) with pleural abnormalities]. In patients with pneumonia, asthmatic bronchitis, and asthma, the percentages of those patients with a positive LUS were 100%, 57.7%, and 0%, respectively. Of note, patients with a positive LUS were associated with an increased need for hospital admission (30.8% vs. 2.4%, *p* = 0.001), administration of oxygen therapy (14.6% vs. 0%, *p* = 0.009), oral corticosteroids (84.6% vs. 19.0%, *p* < 0.001), and antibiotics (88.5% vs. 11.9%, *p* < 0.001); and a higher median value of PRAM score (4.0 (2.0–7.0) vs. 2.0 (1.0–5.0); *p* < 0.001).

**Conclusions:**

Our findings would suggest the use of LUS as a safe and cheap tool used by clinicians to define the diagnosis of school-aged children with wheezing of unknown causes.

## Introduction

Symptoms and signs frequently associated with asthma exacerbation, such as shortness of breath, chest tightness, coughing, and wheezing, are common in respiratory tract infections, such as pneumonia, asthmatic bronchitis, or bronchiolitis (1). When Polymerase Chain Reaction is used to supplement or instead of traditional techniques, viruses have been found in approximately 80% of wheezing episodes in school-aged children (2). To our knowledge, there is limited information available on fast and safe bedside tools that could assist clinicians in establishing whether the pathological process underlying cases of wheezing is due to asthmatic exacerbation, asthmatic bronchitis, or asthma/pneumonia (3). Lung ultrasound (LUS) is a non-invasive, non-ionizing radiation tool that could help to differentiate between various pathological processes by the interplay between air, fluid, and pleurae (4). Despite advances in asthma management, acute exacerbations continue to be challenging in childhood (5). Previous studies have indicated that asthma is still often over-diagnosed in children, mostly at an earlier age (6, 7). This is probably due to the inaccuracy of symptoms and signs alone. In a retrospective study of all 652 children who received the diagnosis of asthma or were treated as having asthma, only 105 children had a diagnosis confirmed with spirometry (6). Although spirometry is the most common asthma diagnostic test used in clinical practice, the lack of standardization between the guidelines and its underuse in primary care complicates the challenge (8). Methods of lung imaging that are applicable to asthma research are now highly developed (9). Indeed, a relatively recent cross-sectional study involving 94 children aged <2 years with wheezing and respiratory infections found that LUS was useful in determining their underlying conditions (10). However, the authors recruited only pre-school-aged children, for whom the diagnosis of asthma would be more difficult. Limited and contrasting data exist on the potential use of LUS in children with wheezing due to the differences in study design, the timing of the sampling, and the lack of homogeneity in the population (10–12). No studies have characterized the potential use of LUS in school-aged children with wheezing. Hence, our primary aim was to characterize LUS in children aged >5 years old with wheezing and symptoms suggestive of respiratory tract infections. As our secondary aim, we investigated the clinical course and healthcare resources for patients with positive LUS findings as.

## Materials and methods

### Study design, setting, and population

We carried out a cross-sectional study in the Pediatric Allergy and Pulmonology Unit of the Department of Pediatrics, University of Chieti, Italy, during the period from November 2018 through December 2019. A pediatric pulmonologist enlisted a sample of consecutive outpatients with wheezing and suggestive signs of acute respiratory infection (cough, blocked nose, shortness of breath, runny nose, fever). We defined wheezing as the presence of a shrill, coarse whistling or rattling sound heard during physical examination of the chest (13). Wheezing is caused by a narrowing or obstruction of the airways due to swelling of the tissues in the airways, bronchospasm, or an accumulation of mucus in the airways (13). Different clinical conditions might cause wheezing upon physical examination (i.e., bronchiolitis, pneumonia, tracheomalacia, asthma, viral lung infections, etc.). Inclusion criteria were: (1) pediatric outpatients aged ≥5 years old with wheezing upon physical examination of the thorax who periodically attended our center for their follow-up, given that they were children with previously physician-diagnosed asthma and inhalant allergies; (2) availability of the pediatric sonographer expert in LUS; (3) no treatment for underlying conditions (i.e., salbutamol or antibiotics) before LUS. We excluded patients with bronco-pulmonary malformation, chest or skeletal deformities, neuromuscular disease, cardiac pathologies, cystic fibrosis, immunodeficiency, or gastroesophageal reflux. Children with no data regarding lung function tests and/or LUS were excluded. It is worth noting that we classified the patients as having: “asthma exacerbation” when they presented only wheezing in the chest and showed abnormal spirometry and/or positive bronchodilator tests (TBD) as per GINA 2021 guidelines (14); “asthma/pneumonia” when they had monolateral crackling sounds in addition to wheezing in the chest and clinical signs, such as fever, tachypnea, or reduced oxygen saturation (15); “asthmatic bronchitis” when they showed bilateral diffusing crackling sounds in addition to wheezing in the chest. Written, informed consent was obtained from parents or legal representatives of all children and adolescents (aged >12 years); in addition, the adolescents were also asked to give their consent. The study was approved by the local Ethical Committee of the University of Chieti (protocol number 2139), and it was conducted in compliance with ethical principles based on the Declaration of Helsinki.

All subjects underwent spirometry and BDT. An expert sonographer, blinded to all subject characteristics, clinical course, and the study pediatrician's diagnosis, performed an LUS after spirometry and before BDT. Similarly, the study pediatrician who decided on the therapy and the hospital admission was blinded to LUS findings. The severity of acute respiratory symptoms in pediatric patients was performed using a validated clinical score, such as the Pediatric Respiratory Assessment Measure (PRAM) (9, 16). The patients were assigned to three severity classes: mild (0–3), moderate (4–7), and severe (8–12), based on the oxygen saturation, suprasternal retractions, scalene muscle contraction, pulmonary air entry, and wheezing. A research assistant collected data from the electronic medical records for the patients' demographics, course of treatment during the pediatric visit, and standardized data collection form on which the physician indicated the presence or absence of specific clinical findings (e.g., wheezing, respiratory distress, retractions, grunting) at the time of their first clinical assessment. As our primary outcome, we characterized LUS in our sample. As our secondary outcome, we compared the clinical course and hospital resources provided to patients with a positive LUS with those provided to patients with a negative LUS.

### Study point-of-care lung ultrasound protocol

Our expert sonologist was certified as an independent practitioner by the Italian Ultrasound Society in Medicine and Biology (SIUMB) and had more than 7 years of experience with point-of-care ultrasound. Accessible lung fields were visualized using commercial Ultrasound Sonography (Samsung HM70A, Republic of Korea, 2013) equipped with a 3 to 16 MHz linear probe. As described by Copetti and Cattarossi (17), the probe was placed perpendicular and parallel to the ribs to view the intercostal spaces. The sonographer slowly examined the chests of the patients while seated, upright, or in the parent's holding position (18). Each hemithorax was divided into six segments: anterior (parasternal to anterior axillary line), lateral (anterior to posterior axillary line), and posterior (posterior axillary line to paravertebral), with each of these three subdivided into superior and inferior segments. Every visualized section of the lung was assessed for normal or abnormal echogenic appearance. If a zone was considered abnormal, the sonographer recorded the specific findings encountered: the absence of lung sliding (the absence of respiration movement between the visceral and parietal pleura); ≥3 B-lines—laser-like vertical hyperechoic reverberation artifacts that arise from the pleural line to the bottom of the image; pleural effusion—anechoic space between the visceral and parietal pleura to the bases of the lungs; micro-consolidation and macro-consolidation—pleural-echo-poor or tissue-like region, respectively less and greater than one centimeter; pleural line irregularity—irregular, thickened, or fragmented pleural line. The LUS was defined as positive in the presence of one or more of the aforementioned findings in any of the subject's lung zones.

### Spirometry

According to the American Thoracic Society/European Respiratory Society guidelines (19), lung function was assessed by flow/volume curves (VyntusTM, Jaeger® IM PRO, Carefusion, Germany 234 GmbH). In an upright position with a nose-block clip applied, the patients underwent a spirometric examination for three consecutive technically acceptable maneuvers. The spirometric parameters evaluated were FEV_1_, forced vital capacity (FVC), FEV_1_/FVC ratio, peak expiratory flow (PEF), and Maximum expiratory flow (MEF) rate at 25% of vital capacity (VC), MEF at 50% of VC, MEF at 75% of VC. The lung function parameters were converted into sex-, height-, age-, and ethnicity-adjusted *z*-scores according to the Global Lung Initiative (GLI) reference data (17). FEV_1_ *z*-score < −1.64 was defined as positive for obstructive airway disease (20). A Lung function evaluation was performed by a pediatric pulmonologist and the best spirometric measurements were considered for statistical calculations. Our maximal tolerated variability for the three lung function measurements was less than 10%, as reported by Pellegrino et al. (21).

### Statistical analysis

Continuous data were expressed as mean ± standard deviation (SD), and categorical data were presented as counts and percentages. The positive spirometry and LUS results (and each of their patterns) were expressed as frequencies and proportions overall. We used an unpaired *t*-test or Mann-Whitney test for continuous variables and a chi-squared (*χ*^2^) test or Fisher's exact test for categorical variables. The clinical diagnosis was based on the presence of acute respiratory symptoms (fever, cough, tachypnea, etc.) and wheezing with or without localized or diffused crackles upon chest auscultation. The level of statistical significance was set at *p* < 0.05. All analyses were run in R 3.6.2 [Language and Environment for Statistical Computing. R Core Team, R Foundation for Statistical Computing, Vienna, Austria, 2019; (https://www.R-project.org/)].

## Results

**Figure 1:**
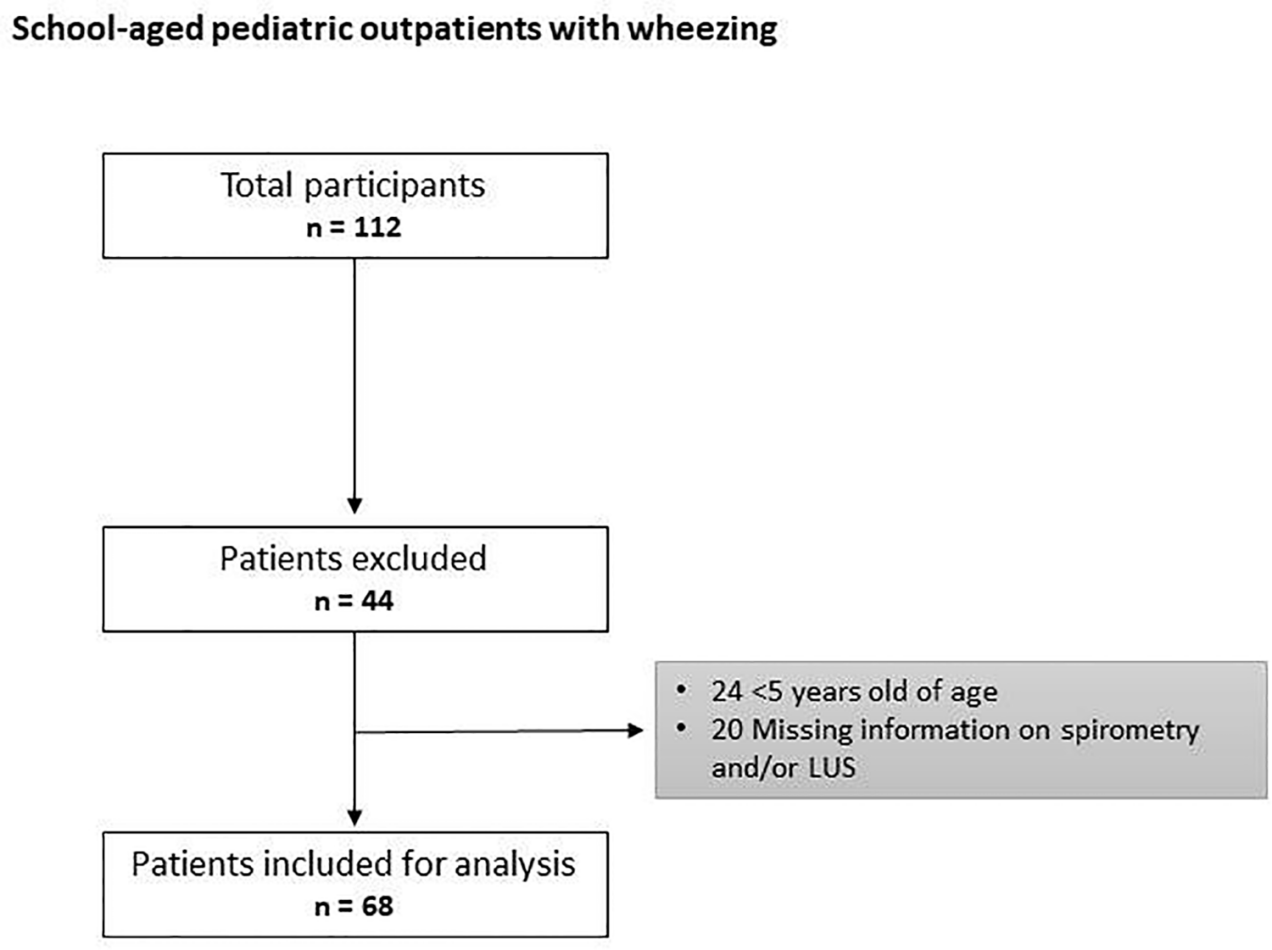
Flow-chart of the study. LUS, lung ultrasound.

The flow chart of the study is shown in Figure 1.

**Figure 2:**
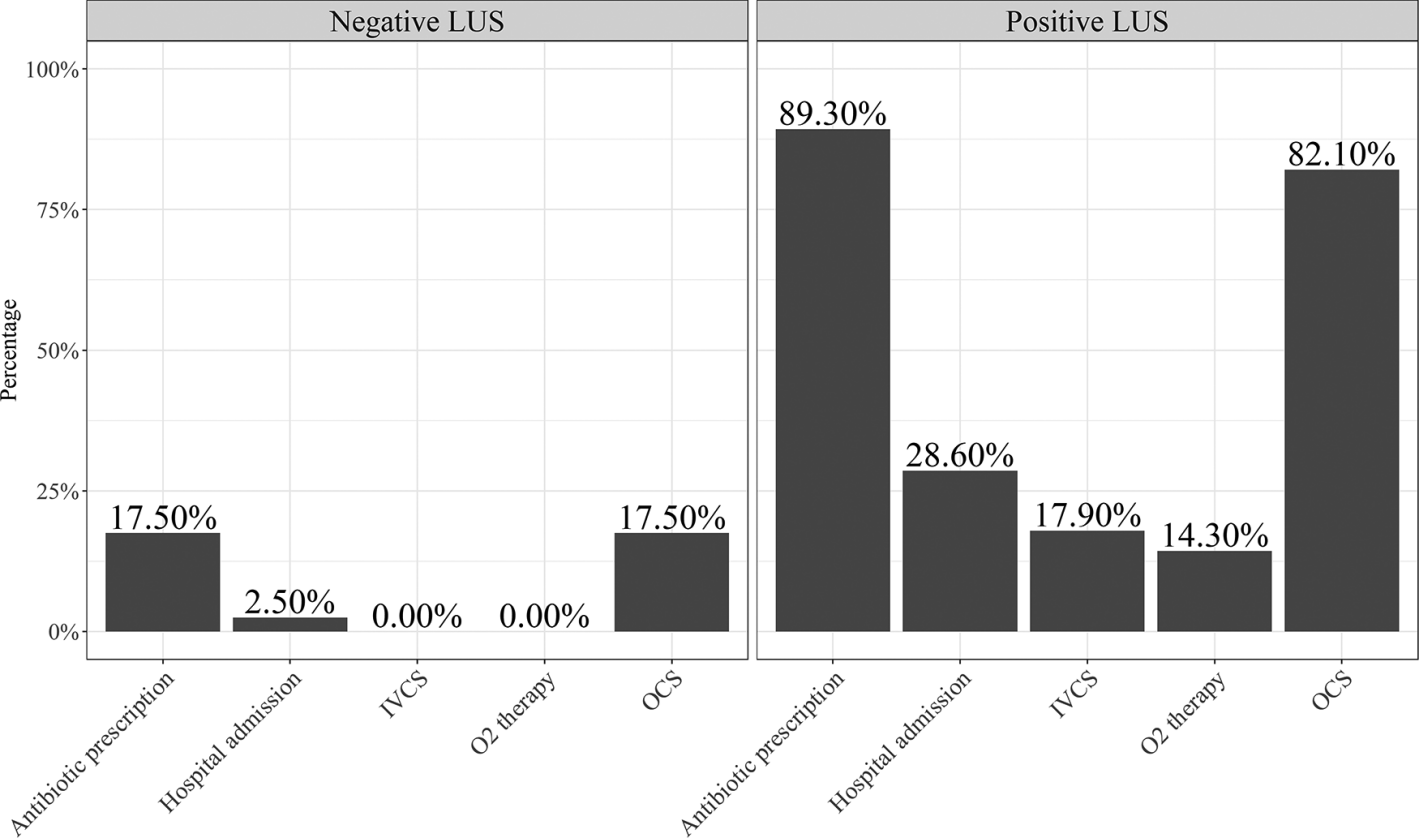
Description of the clinical diagnosis of patients with wheezing. LUS, lung ultrasound.

We enrolled 68 consecutive outpatients (mean age of 9.9 ± 3.4 years). The characteristics of the patients are shown in Table 1. The percentages of patients studied at our center who had been previously diagnosed with asthma and inhalant allergies were 85.3% and 82.4%, respectively. Forty-eight (70.6%) patients controlled their asthma symptoms by taking inhaled corticosteroids and fifty-six (82.4%) controlled their allergy symptoms by taking antihistamines. Forty-five (66.2%) patients showed a mild respiratory episode and twenty-three (33.8%) showed a moderate one based on PRAM scores. The clinical diagnoses of our patients are described in Figure 2. The LUS was positive in 38.2% (26/68) of the patients, 12 of whom (46.1%) presented multiple B-lines, 14 (53.8%) micro-consolidation, 10 (38.5%) macro-consolidation, 9 (34.6%) pleural thickening, and 13 (50.0%) pleural effusion. Twenty-six (38.2%) patients showed an obstructive pattern to spirometry and ten patients (14.7%) presented a positive response to BDT, identifying an asthma exacerbation. Few patients (7.4%) underwent a chest x-ray (CXR) to rule out any potential complications in consideration of the severe clinical picture of those children. CXR was positive for pneumonia in three patients. The percentages of patients with a positive LUS among those with asthma/pneumonia, asthmatic bronchitis, and asthma were: 100% (11 patients), 57.7% (15 patients), and 0% (0 patients), respectively. Additionally, antibiotics were used more frequently in the asthma/pneumonia group compared with the asthmatic bronchitis group and asthma group (90.9%, 65.4%, and 3.2%, respectively). Similarly, oral and intravenous corticosteroids were administered more frequently in the asthma/pneumonia group than the other two groups (100%, 46.2%, 22.6%; and 36.4%, 3.8%, 0.0%, respectively). Oxygen (O_2_) therapy was delivered more frequently in the asthma/pneumonia group than in the other two groups (27.3, 3.8%, and 0.0% respectively). It is worth noting that the median value of the PRAM score (severity index) was higher in the asthma/pneumonia group compared with the asthmatic bronchitis group and asthma group. The description of LUS characteristics and the follow-up conducted by clinical groups are presented in Table 2.

**Table 1:** General characteristics of our study population.

	All Patients (*n* = 68) *n* (%)
**General characteristics**	
Sex (%)	
Male	47 (69.1)
Female	21 (30.9)
Age (years)	
	9.9 (3.4)
Height (cm)	
	138.0 (18.2)
Weight (Kg)	
	38.8 (16.6)
Race (%)	
Caucasian	62 (91.2)
Other	6 (8.8)
**Mother and birth charateristics**	
Mode of delivery (%)	
Vaginal-assisted	43 (63.2)
Caesarean	25 (36.8)
Gestational age at birth (weeks)	
** **	39.1 (1.8)
Birth Weight (Kg)	
** **	3.4 (0.5)
Smoking during pregnancy (%)	
No	63 (92.6)
Yes	5 (7.4)
RDS at birth (%)	
No	64 (94.1)
Yes	4 (5.9)
nCPAP at birth (%)	
No	65 (95.6)
Yes	3 (4.4)
Intubation at birth (%)	
No	67 (98.5)
Yes	1 (1.5)
O_2_-therapy at birth (%)	
No	64 (94.1)
Yes	4 (10.9)
**Parental history**	
History of parental allergies (%)	
No	31 (45.6)
Yes	37 (54.4)
History of parental asthma (%)	
No	49 (72.1)
Yes	19 (27.9)
**Medical history**	
Physician-diagnosed asthma (%)	
New diagnosis	10 (14.7)
Previous diagnosis	58 (85.3)
Presence of allergy^a^	
No	12 (17.6)
Yes	56 (82.4)
**Therapy**	
Inhaled corticosteroid (%)	
No	20 (29.4)
Yes	48 (70.6)
Antihistamine (%)	
No	17 (25.0)
Yes	51 (75.0)
Nasal corticosteroid (%)	
No	38 (55.9)
Yes	30 (44.1)
**Acute respiratory symptoms severity**	
PRAM score (%)	
Mild (1-3)	45 (66.2)
Moderate (4-7)	23 (33.8)
Severe (8-12)	0 (0.0)

Data are presented as means (SD) and valid percentages (absolute numbers). No signiﬁcant difference when *p* > 0.05. Signiﬁcant *p* values are marked in bold. RDS, respiratory distress syndrome; nCPAP, nasal continuous positive airway pressure; O_2_, oxygen therapy; PRAM, pediatric respiratory assessment measure

^a^ The presence of allergy was based on skin prick test results.

**Table 2:** Description of LUS characteristics and follow-up by clinical groups.

	Asthma/ Pneumonia (*n* = 11)	Asthmatic bronchitis (*n* = 26)	Asthma (*n* = 31)
Micro-consolidation (%)	4 (6.4)	10 (38.5)	0 (0.0)
Macro-consolidation (%)	7 (63.6)	3 (11.5)	0 (0.0)
Pleural effusion (%)	6 (54.6)	7 (26.9)	0 (0.0)
Pleural thickning (%)	3 (27.3)	6 (23.1)	0 (0.0)
B-lines (%)	6 (54.6)	6 (23.1)	0 (0.0)
Antibiotics (%)	10 (90.9)	17 (65.4)	1 (3.2)
oCS (%)	11 (100)	12 (46.2)	7 (22.6)
ivCS (%)	4 (36.4)	1 (3.8)	0 (0.0)
CXR (%)	4 (36.4)	0 (0.0)	1 (3.2)
O_2_ therapy (%)	3 (27.3)	1 (3.8)	0 (0.0)
Hospital admission (%)	5 (45.5)	3 (11.5)	1 (3.2)
PRAM-score	5.0 (2.0–7.0)	3.5 (1.0–7.0)	2.0 (1.0–4.0)

Data are expressed as absolute numbers and percentages or median and range 5%–95%. O_2_, oxygen; oCS, oral corticosteroids; ivCS, intravenous corticosteroids; CXR, chest x-ray; PRAM, pediatric respiratory assessment measure.

Patients with a positive LUS showed an increased need for hospital admission (30.8% vs. 2.5%, *p* = 0.001), administration of O_2_ therapy (15.6% vs. 0.0%, *p* = 0.009), oral corticosteroids (84.6% vs. 19.0%, *p* < 0.001), and antibiotics (88.5% vs. 11.9%, *p* < 0.001) and higher median value of PRAM score (4.0 (2.0–7.0) vs. 2.0 (1.0–5.0); *p* < 0.001) than patients with a negative LUS. In addition, patients with a positive LUS showed a lower mean FEV_1_ z score value than those with a negative LUS (−1.90 (1.9) vs. −0.60 (1.6); *p* = 0.004).

The clinical course of the patients according to the LUS findings is shown in Table 3.

**Table 3:** Clinical course and hospital resource use of the patients with positive lung ultrasound.

	Patients with positive LUS (*N* = 28) *n* (%)	Patients with negative LUS (*N* = 40) *n* (%)	*p* value
Hospital admission			***0***.***002***
No	20 (71.4)	39 (97.5)	* *
Yes	8 (28.6)	1 (2.5)	* *
Antibiotic prescription			***<0***.***001***
No	4 (10.7)	33 (83.5)	* *
Yes	25 (89.3)	7 (17.5)	* *
Molecule type of antibiotics			***<0***.***001***
azithromycin	19 (67.9)	5 (12.5)	* *
clarithromycin	5 (17.8)	2 (5.6)	*** ***
Amoxicillin/clavulanic acid + clarithromycin	0 (0.0)	1 (2.5)	*** ***
No antibiotics	4 (14.3)	32 (80.0)	*** ***
OCS need			***<0***.***001***
No	5 (17.9)	33 (82.5)	* *
Yes	23 (82.1)	7 (17.5)	* *
IVCS need			***0***.***005***
No	23 (82.1)	40 (100)	* *
Yes	5 (17.9)	0 (0.0)	* *
O_2_ therapy need			* *
No	24 (85.7)	40 (100)	***0***.***010***
Yes	4 (14.3)	0 (0.0)	* *

Values are absolute numbers (percentages). *N*, number; LUS, lung ultrasound; OCS, oral corticosteroids; IVCS, intravenous corticosteroids; O_2_, oxygen.

Bold formatting to values where the *p*-value is <0.05.

*p* value from post-hoc test for Chi-squared test.

Additionally, we found that there was a significant difference in LUS findings between the asthma/pneumonia group and the asthmatic bronchitis group. In particular, macro-consolidation LUS findings were more frequent in the asthma/pneumonia group than the asthmatic bronchitis group (63.6% vs. 11.5%; *p* value = 0.001). No differences were found in other LUS findings (Table 4).

**Table 4:** Description of LUS abnormalities by asthma/pneumonia group and asthmatic bronchitis group.

	Asthma/ Pneumonia (*n* = 11)	Asthmatic bronchitis (*n* = 26)	*p* value
Micro-consolidation (%)	4 (36.4)	10 (38.5)	0.904
Macro-consolidation (%)	7 (63.6)	3 (11.5)	**0** **.** **001**
Pleural effusion (%)	6 (54.5)	6 (23.1)	0.062
Pleural thickning (%)	3 (27.3)	3 (23.1)	0.786
B-lines (%)	6 (54.5)	6 (23.1)	0.062

Data are expressed as absolute numbers and percentages. O_2_, oxygen; oCS, oral corticosteroids; ivCS, intravenous corticosteroids; CXR, chest x-ray.

Bold formatting to values where *p*-value is < 0.05.

*p*-values from Pearson's Chi-squared test.

## Discussion

The main finding of our study was a negative LUS pattern (presence of A-lines plus lung sliding) among patients with only asthma. Abnormalities in the LUS were found in all patients with a clinical diagnosis of asthma/pneumonia. Children with asthma/pneumonia or with asthmatic bronchitis showed a more severe clinical condition compared with children with asthma, as expressed by higher PRAM score values. In addition, we observed that patients with a positive LUS had a worse clinical course compared with those with a negative LUS, suggesting the potential use of LUS as an efficient follow-up tool in more severe lung diseases. From a clinical point of view, this aspect is interesting as the treatments for asthma and pneumonia are very different. In clinical practice, this would reduce the overprescribing of ineffective antibiotics and systemic corticosteroids or predict hospital admission and the use of O_2_ therapy. Considering this, LUS could be used as a bedside tool for clinicians to address the underlying health issue of patients with wheezing of unknown cause. To date, only two preliminary studies have tried to characterize LUS patterns in children with wheezing. Varshney et al. (10) describe the LUS pattern in children aged <2 years with respiratory tract infections and wheezing and report that none of the patients with a final diagnosis of asthma had a positive LUS. In an adult population with undifferentiated respiratory distress, Lichtenstein et al. (11) show that predominant A-lines plus lung sliding are consistent with the diagnosis of asthma with 89% sensitivity and 97% specificity. In a recent consensus obtained from a panel of 13 experts from five Polish tertiary pediatric centers, LUS was considered useful for diagnosing community-acquired pneumonia (CAP) in children with a level of evidence A, whereas normal LUS findings in children with suspected lower respiratory tract infections (LRTIs) significantly reduced the probability of diagnosing CAP (22). Volpicelli et al. (3) also state that where pneumonia is suspected, positive LUS excludes the need to perform CXR, supporting the suggestion that LUS should be considered first for pediatric patients suspected of CAP who require diagnostic imaging (3). Several studies that were also carried out on a pediatric population show that LUS not only has greater sensitivity and specificity than CXR (23–25) but is also able to distinguish inflammatory/infectious consolidations from atelectasis (23, 24) and distinguish an inflammatory picture of viral origin from that of bacterial origin.

In contrast, Dankoff et al. (26) observe that 45% of pediatric patients (mostly of age <6 years) with an asthmatic background had a positive LUS pattern during asthmatic exacerbations. However, the clinical course was characterized by antibiotics, CXR scans consistent with pneumonia, and the age of the patients involved to suppose whether pediatric patients were affected by a respiratory infection rather than asthma. The same doubts were also expressed in a letter to the editor by Longacre et al. (27). Recently, De Rose et al. (28) described LUS abnormalities that were present in a case series characterized by children with severe uncontrolled asthma. The authors state that LUS abnormalities were observed in patients with severe asthma and that different imaging patterns depend on the severity and control of the asthma. Specifically, children with uncontrolled asthma showed the presence of lung atelectasis, which resolved slowly after weeks of therapy to treat acute attacks and the beginning of adequate preventive therapy. On the contrary, in children with asthma that is well controlled by adequate preventive therapy, LUS was not highly positive, showing slight sonographic signs of interstitial syndrome.

We would suggest that the information derived from LUS could complement what is contained in clinical evaluation, improving the treatment of children with wheezing. In clinical practice, a seven-year-old child with fever, tachypnea, wheezing, non atopic background and a positive LUS would address the clinician far from an episode of asthma and more toward a LRTI, whereas a child with a negative LUS and presence of inhalant allergy would address the clinician less likely towards a LRTI. This aspect would avoid the “over-prescription” of antibiotics and CXRs.

Indeed, in a recent survey, Patel et al. (29) investigate the behavior of practicing physicians in ordering CXRs in pediatric patients presenting with their first episode of wheezing to an emergency department (ED) and the factors that influence their practice. The authors found that 30% of ED physicians would have routinely requested CXR in pediatric patients with their first episode of wheezing (29). Importantly, in this latter study, the factors influencing that practice were primary residency and fellowship training, resident supervision, and years of independent practice. Therefore, CXR in pediatric patients with wheezing is still a critical issue, mostly because of the poor education of physicians, although literature supports its limited use.

To the best of our knowledge, we carried out the first study to investigate LUS patterns in school-aged children with wheezing. Although our aim was to propose the application of LUS in the diagnostic treatment of pediatric patients, there are some limitations, which need to be discussed. First of all, the reliability of LUS is dependent on the experience of the ultrasonographer. In the present study, LUS scans were performed by a single experienced operator because, in our Department of Pediatrics, the investigator was the only pediatric sonographer expert in LUS. It is reasonable to hypothesize that similar results from our sonographer might be biased, even though he was blinded to all patient characteristics, the clinical course, and the interpretations of the study's pediatric pulmonologist prior to each LUS examination. In addition, we did not perform viral tests or CXR in all participants in order to better establish the diagnosis underlying the pathological process.

However, the following doubts remain: is a negative LUS pattern always consistent with only asthma? Might asthmatic airway alterations (i.e., airway edema, mucus plugs, secretions) lead to visible LUS parenchymal abnormalities (inflammation of the pulmonary cortex, sub-segmental atelectasis, or hypoventilation)? Is the characterization of LUS uniform between children and adults during an asthmatic exacerbation (different triggers, different inflammation/remodeling ratio)? The description of inflammatory changes within the alveolar tissue of asthma patients (30, 31) might query the certainty that asthma is an “airways-only” disease (32).

However, caution is needed in interpreting the preliminary findings of our study and further studies on a larger scale are necessary. Well-conducted, randomized controlled studies are necessary to demonstrate that better health outcomes occur when LUS is integrated as part of the diagnostic assessment. Similarly, prospective longitudinal studies in well-characterized subgroups of asthma patients, including invasive procedures (i.e., bronchoscopy, BAL, or transbronchial biopsy), are required in order to determine the potential changes in the lung parenchyma during asthmatic episodes and to better study the structure-function relationships.

### Take-home message

LUS could be used as a bedside tool for clinicians to define, in addition to clinical evaluation, the diagnosis of school-aged children with wheezing of unknown causes. In addition, we would also point out that the use of LUS in children with wheezing might avoid repeated CXR in both asthma and respiratory infections. However, well-conducted, randomized controlled studies are necessary to demonstrate that better health outcomes occur when LUS is integrated as part of the diagnostic assessment.

## Data Availability

The raw data supporting the conclusions of this article will be made available by the authors, without undue reservation.
